# Acute clinical evaluation for syndesmosis injury has high diagnostic value

**DOI:** 10.1007/s00167-022-06989-2

**Published:** 2022-05-04

**Authors:** Thomas P. A. Baltes, Omar Al Sayrafi, Javier Arnáiz, Maryam R. Al-Naimi, Celeste Geertsema, Liesel Geertsema, Louis Holtzhausen, Pieter D’Hooghe, Gino M. M. J. Kerkhoffs, Johannes L. Tol

**Affiliations:** 1 Research Department, Aspetar Orthopaedic and Sports Medicine Hospital, Doha, Qatar; 2 Department of Orthopaedic Surgery, Amsterdam UMC, University of Amsterdam, Amsterdam Movement Sciences, Amsterdam, The Netherlands; 3 Academic Center for Evidence-Based Sports Medicine (ACES), Amsterdam UMC, Amsterdam Movement Sciences, Amsterdam, The Netherlands; 4 Amsterdam Collaboration for Health and Safety in Sports (ACHSS), AMC/VUmc IOC Research Center, Amsterdam, The Netherlands; 5 Department of Sports Medicine, Aspetar Orthopaedic and Sports Medicine Hospital, Doha, Qatar; 6 Department of Radiology, Aspetar Orthopaedic and Sports Medicine Hospital, Doha, Qatar; 7 Section Sports Medicine, Faculty of Health Sciences, University of Pretoria, Pretoria, South Africa; 8 Department of Orthopaedic Surgery, Aspetar Orthopaedic and Sports Medicine Hospital, Doha, Qatar

**Keywords:** Ankle sprain, Syndesmosis, Physical examination

## Abstract

**Purpose:**

To determine the diagnostic value of injury history, physical examination, six syndesmosis tests and overall clinical suspicion for syndesmosis injury.

**Methods:**

All athletes (> 18 yrs) with an acute ankle injury presenting within 7 days post-injury were assessed for eligibility. Acute ankle injuries were excluded if imaging studies demonstrated a frank fracture or 3 T MRI could not be acquired within 10 days post-injury. Standardized injury history was recorded, and physical examination was performed by an Orthopaedic Surgeon or Sports Medicine Physician. Overall clinical suspicion was documented prior to MRI. Multivariate logistic regression was used to determine the association between independent predictors and syndesmosis injury.

**Results:**

Between September 2016 and July 2019, a total of 150 acute ankle injuries were included. The median time from injury to acute clinical evaluation was 2 days (IQR 2). Prior to clinical evaluation, the median patient reported Visual Analog Scale for pain was 8/10 (IQR 2). Syndesmosis injury was present in 26 acute ankle injuries. An eversion mechanism of injury had a positive LR 3.47 (CI 95% 1.55–7.77). The squeeze tests had a positive LR of 2.20 (CI 95% 1.29–3.77) and a negative LR of 0.68 (CI 95% 0.48–0.98). Overall clinical suspicion had a sensitivity of 73% (CI 95% 52–88) and negative predictive value of 89% (CI 95% 78–95). Multivariate regression analyses demonstrated significant association for eversion mechanism of injury (OR 4.99; CI 95% 1.56–16.01) and a positive squeeze test (OR 3.25; CI 95% 1.24–8.51).

**Conclusions:**

In an acute clinical setting with patients reporting high levels of ankle pain, a negative overall clinical suspicion reduces the probability of syndesmosis injury. Eversion mechanism of injury and a positive squeeze test are associated with higher odds of syndesmosis injury.

**Level of evidence:**

Level III.

**Supplementary Information:**

The online version contains supplementary material available at 10.1007/s00167-022-06989-2.

## Introduction

Acute ankle sprains are among the most common sport-related injuries [7]. Sprains can affect the lateral ligaments, medial ligaments and the syndesmosis ankle ligaments. Complete rupture of the lateral ligaments is observed most commonly (40%) [19]. Within 2 days after injury, physical examination for diagnosing lateral ligament injury has a sensitivity of 71% and a specificity of 33% [6]. Delayed physical examination 5 days post-injury increases the sensitivity (96%) and specificity (84%). Syndesmosis injury is observed less frequently (20%) and is notoriously difficult to diagnose [19]. In the acute setting, the diagnosis of syndesmosis injury primarily relies on clinical evaluation [23].

Conflicting reports on the diagnostic value of physical examination have been published [15]. Two recent high-quality studies investigated the diagnostic value of clinical tests for partial or complete discontinuity of the syndesmosis using MRI [8, 22]. In a cohort of 96 patients presenting to an emergency department within 24 h after an acute ankle sprain, sensitivity (14–56%) and specificity (48–83%) of syndesmosis tests were reported as insufficient [8]. In a cohort of 87 athletes examined within 7 days after a suspected syndesmosis injury, tenderness of the anterior syndesmosis was reported to have the highest sensitivity (92%) and the squeeze test was found to have the highest specificity (88%) [22]. The heterogeneity in reported diagnostic values might be explained by the timing of physical examination and higher pre-test probability in the latter study. A study investigating the diagnostic value of injury history, physical examination, and overall clinical suspicion in athletes with an acute ankle injury is therefore warranted.

The aim of this study was to determine the diagnostic value of injury history, clinical findings, six syndesmosis tests (including the combination of these variables) and overall clinical suspicion for partial or complete discontinuity of the syndesmosis. The hypothesis is that in the acute setting, the diagnostic value of injury history, physical examination and overall clinical suspicion are sufficient to detect syndesmotic involvement in athletes with an acute ankle injury.

## Materials and methods

### Participants

Ethics approval was acquired from the Anti-Doping Lab Qatar Review Board (IRB No. F2016000153). Written informed consent was obtained from all athletes at time of inclusion. Between September 2016 and July 2019, athletes with acute ankle injuries were recruited for this prospective cohort study. All athletes presenting to the outpatient department of Aspetar Orthopaedic and Sports Medicine Hospital within 7 days after an acute ankle injury were assessed for eligibility. Inclusion criteria were: acute ankle injuries in adult athletes (≥ 18 yrs), participating in sports at a professional or recreational level. Ankle injuries were excluded if imaging demonstrated a frank fracture or if the 3 T MRI study could not be acquired within 10 days post-injury.

### Clinical setting

A daily clinic for athletes with acute sports injuries is organized by Aspetar Orthopaedic and Sports Medicine Hospital. Patients can be seen by an Orthopaedic Surgeon or Sports Medicine Physician without the requirement of a referral. This results in a high volume of patients of which the majority presents within 24 h post-injury. The main language spoken by the medical staff at our institution is English; therefore, during consultation a nurse is present to provide Arabic translation.

### Injury history and physical examination

An Orthopaedic Surgeon or Sports Medicine Physician recorded injury history and performed physical examination. Findings were recorded on a standardized reporting form. Injury history included: (1) Injury [new/recurrent] (2) Occasion [game/training/non-sports injury], (3) Contact [contact/non-contact], (4) Mechanism of injury [inversion/eversion/external-rotation/internal-rotation], (5) Perceived presence of swelling [yes/no], (6) Perceived ankle instability [yes/no], (7) Sensation of pain radiating up the leg [yes/no]. Physical examination included; (1) Presence of hematoma [yes/no] (2) Tenderness to palpation [lateral/medial/anterior/posterior] (3) Tenderness length over the syndesmosis [in cm], (4) Ability to walk normally [yes/no], (5) Ability to walk on toes [yes/no], Ability to walk on heels [yes/no], (6) Passive range of motion in dorsal flexion, plantar flexion, inversion and eversion [full/restricted/painful], (7) Presence of swelling [yes/no], (8) Swelling site [laterally/medially/anterior/posterior/syndesmosis].

### Syndesmosis tests

The physicians performing the syndesmosis tests were provided with a short description of the clinical tests. No calibration session was organized prior to initiation of this study, to reflect a true clinical setting. The standardized clinical examination included a total of six syndesmosis tests: palpation of the AITFL [23]; squeeze test [10, 23], weight-bearing dorsiflexion external rotation (WB DF ER) test, non-weight-bearing dorsiflexion external rotation (NWB DF ER) test [16, 23], fibular translation test [17] and the Cotton test [3] (Supplementary appendix). Tests were considered positive if they provoked pain over the distal tibiofibular joint or if the examiner noticed instability of the ankle syndesmosis (Cotton test) [3, 10, 16, 17]. If the patient was unable to weight-bear (WB DF ER) or if a clinical test was too painful to be completed fully (Squeeze test, NWB DF ER test, Fibular translation test) they were considered positive (Cotton test excluded).

### Overall clinical suspicion of syndesmosis injury

Clinical suspicion was based on the physicians’ overall interpretation of injury history, physical examination, and syndesmosis tests. Clinical suspicion was recorded using the modified West Point grading system[4]: partial rupture of the Anterior Inferior Tibiofibular Ligament (AITFL) with a stable syndesmosis (Grade I); complete rupture of the AITFL and injury to the Interosseous Ligament (IOL) with a stable syndesmosis (Grade IIa); complete rupture of the AITFL, IOL and the Posterior Inferior Tibiofibular Ligament (PITFL) or Deltoid ligaments with dynamic instability of the syndesmosis (Grade IIb) and complete disruption of the syndesmosis with frank diastasis (Grade III). Overall clinical suspicion for syndesmosis injury was considered positive when the modified West Point grading system was scored grade I or higher.

### Reference standard

In this study, MRI was used as reference standard. With a sensitivity of 100% and specificity of 93% for injury of the AITFL, MRI has demonstrated to be a valuable alternative to arthroscopy [24]. Patients underwent MRI scans using a wide-bore 3.0-T MRI system (GE Discovery, GE Healthcare, Chicago, Illinois, United States) with an 8-channel receive only Foot & Ankle array (Invivo, Philips Healthcare, Best, The Netherlands). In the sagittal plane T1-weighted and Proton-Density Fat-Saturated [PD-FS] sequences were obtained, axial T2-weighted and PD-FS sequences were acquired and in the coronal plane PD-FS sequences were obtained [1].

### Grading of syndesmosis ligaments

The obtained MR scans were graded by two musculoskeletal radiologists (J.A. & M.A.) with 11 and *3* years of experience in MSK-imaging, respectively.

Acute injuries of the AITFL, IOL and PITFL were graded according the four grade Schneck grading system[20]: normal (Grade 0); low-grade sprain (Grade 1: peri-ligamentous high signal/edema on proton density-weighted sequences and no discontinuity of fibers); partial discontinuity (Grade 2: partial discontinuity but preserved remnant fibers) and complete discontinuity (Grade 3).

Due to limited interrater and intrarater reliability for grading of the individual syndesmosis ligaments according the Schneck grading system (K 0.37–0.89), diagnostic disagreements between the radiologists were resolved in a consensus meeting [1].

### Evaluation of MRI-findings

In this study, we evaluated the diagnostic value of injury history, physical examination, syndesmosis tests and clinical suspicion for partial or complete discontinuity of the AITFL and/or IOL and/or PITFL. The individual syndesmosis ligament (AITFL/IOL/PITFL) with the highest grade of injury was used as reference standard. For this analysis, grading was dichotomized as: (1) No discontinuity (normal ligament or peri-ligamentous edema) (2) Discontinuity (partial or complete discontinuity).

### Statistical analysis

The diagnostic value of injury history, clinical findings, syndesmosis tests and clinical suspicion were evaluated using the MRI findings. The results of injury history and physical examination were dichotomized if needed; Occasion was dichotomized to (1) game (2) other; Each mechanism of injury was dichotomized to i.e. (1) inversion (2) other mechanism of injury. Tenderness to palpation and presence of swelling were dichotomized per location to i.e. (1) tenderness to palpation laterally (2) no palpation to tenderness laterally and range of motion was dichotomized per direction to i.e. (1) dorsal flexion painful (2) dorsal flexion not painful.

For each variable, a contingency table was created. From the 2 × 2 tables prevalence of positive test results, sensitivity, specificity, positive and negative likelihood ratio’s (LR+ and LR-), positive predictive value (PPV) and negative predictive value (NPV) were calculated, including confidence intervals (CI 95%). For tenderness length an optimal cut-off (Youden’s index = maximal value) was calculated using a ROC curve. For each variable the area under the curve (AUC) was calculated.

To test if a combination of variables could predict the presence of syndesmosis injury, we performed a logistic regression analysis. First, we analyzed the association between each variable and the presence of syndesmosis injury in a univariate logistic regression analysis. Variables with a *p* value < 0.15 were included in a multivariate logistic regression model. Regression analysis was performed using the forward method. Odds ratios for each variable were presented with their corresponding confidence intervals (CI 95%) and statistical significance was set at *p* value < 0.05. Statistical analysis was performed using SPSS software (V.21; IBM Corp). No a priori power analysis was performed for the outcome of this study.

## Results

### Baseline characteristics

**Fig. 1:**
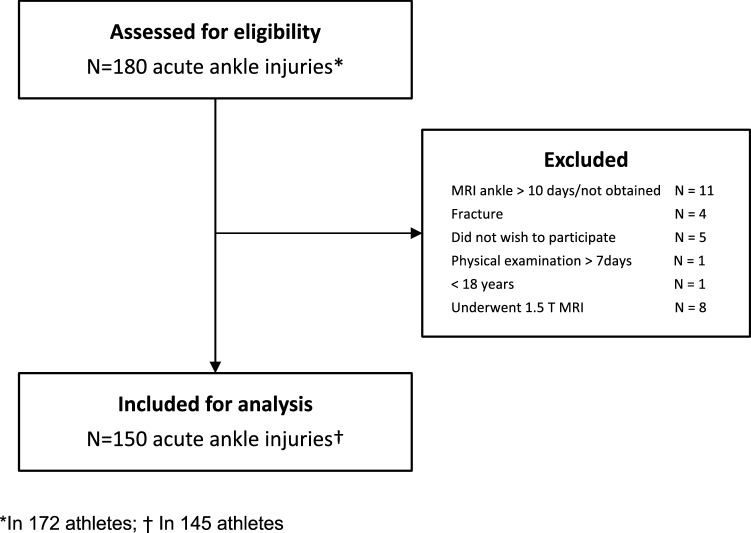
Flowchart of patient inclusion

Between September 2016 and July 2019, a total of 180 acute ankle injuries were assessed for eligibility (Fig. 1). One-hundred-fifty were included in this study. (Flowchart 1) Of the included ankle injuries, four were subsequent injuries of the contralateral ankle and one was a re-injury (> 1 year). Most patients were male (91%). The median age at time of injury was 24 years (IQR 8). The median time from injury to physical examination was 2 days (IQR 2). At presentation, a median patient reported Visual Analog Scale (VAS) for pain of 8/10 (IQR 2) was recorded (in 129 patients). The MR scans were obtained a median 3 days (IQR 3) post-injury. Partial or complete discontinuity of the syndesmosis ligaments was observed in 26 of 150 (17%) acute ankle injuries. Injury of the AITFL was present in 26 acute ankle injuries (17%; 7 partial, 19 complete). The IOL was injured in 20 acute ankle injuries (13%; 8 partial, 12 complete) and the PITFL in 11 acute ankle injuries (7%; 10 partial, 1 complete). Concomitant injuries of the IOL or PITFL were not observed without injury of the AITFL.

### Diagnostic value of injury history, physical examination and overall clinical suspicion

The diagnostic value of injury history and physical examination are detailed in Tables 1 and 2. We found that an external rotation mechanism and eversion mechanism of injury had a positive LR of 4.77 (CI 95% 0.70–32.33) and 3.47 (CI 95% 1.55–7.77), respectively. Length of tenderness [≥ 2.5 cm] had a positive LR of 1.45 (CI 95% 1.02–2.04). Tenderness to palpation over the medial aspect of the ankle had a negative LR of 1.47 (CI 95% 1.06–2.03). The diagnostic values for the syndesmosis tests and clinical suspicion are detailed in Table 3. The squeeze test had the most diagnostic value with a positive LR of 2.20 (CI 95% 1.29–3.77) and a negative LR of 0.68 (CI 95% 0.48–0.98). Clinical suspicion for syndesmosis injury had a sensitivity of 73% and a negative predictive value of 89%.

**Table 1:** Diagnostic accuracy of injury history for injury of the syndesmosis ligaments in 150 athletes with an acute ankle injury. MR imaging was used as reference standard

Clinical history	Positive findings	Sensitivity %	Specificity %	LR+	LR−	PPV %	NPV %	AUC
Injury [recurrent]	37/150 (0.25)	12 (3–31)	73 (64–80)	0.42 (0.14–1.27)	1.22 (1.05–1.41)	8 (2–23)	80 (71–86)	0.42 (0.31–0.53)
Occasion [game]	78/150 (0.52)	58 (37–76)	49 (40–58)	1.14 (0.78–1.65)	0.86 (0.54–1.37)	19 (12–30)	85 (74–92)	0.53 (0.41–0.66)
Contact [contact]	69/138 (0.50)	38 (19–61)	48 (39–57)	0.73 (0.41–1.29)	1.29 (0.91–1.84)	12 (5–22)	81 (70–95)	0.44 (0.34–0.54)
Mechanism of injury								
Inversion	126/150 (0.84)	65 (44–82)	12 (7–19)	0.74 (0.56–0.99)	2.86 (1.50–5.44)	13 (8–21)	63 (41–80)	0.39 (0.26–0.52)
Eversion	19/150 (0.13)	31 (15–52)	91 (84–95)	3.47 (1.55–7.77)	0.76 (0.59–0.98)	42 (21–66)	86 (79–91)	0.61 (0.48–0.74)
External rotation	4/150 (0.03)	8 (1–27)	98 (94–99)	4.77 (0.70–32.33)	0.94 (0.84–1.05)	50 (9–91)	50 (9–91)	0.53 (0.40–0.66)
Internal rotation	6/150 (0.04)	8 (1–27)	97 (91–99)	2.38 (0.46–12.34)	0.95 (0.85–1.07)	33 (6–76)	83 (76–88)	0.52 (0.40–0.65)
Perceived swelling	126/149 (0.85)	85 (64–95)	15 (10–23)	1.00 (0.84–1.20)	1.00 (0.37–2.68)	17 (11–25)	83 (60–94)	0.50 (0.38–0.62)
Perceived instability	37/130 (0.28)	21 (8–43)	70 (60–78)	0.69 (0.30–1.59)	1.13 (0.92–1.40)	14 (5–30)	80 (70–87)	0.47 (0.36–0.57)
Pain radiating up	37/146 (0.25)	26 (11–49)	75 (66–82)	1.04 (0.49–2.20)	0.99 (0.77–1.27)	16 (7–33)	84 (76–90)	0.50 (0.38–0.63)

The prevalence of acute syndesmosis injuries and the diagnostic values of clinical history are presented. All values are presented with 95% confidence interval (95% CI); positive likelihood ratio (LR+); negative likelihood ratio (LR-); positive predictive value (PPV); negative predictive value (NPV)

**Table 2:** Diagnostic accuracy of physical examination for injury of the syndesmosis ligaments in 150 athletes with an acute ankle injury. MR imaging was used as reference standard

Clinical findings	Positive findings	Sensitivity %	Specificity %	LR+	LR−	PPV %	NPV %	AUC
Presence of hematoma	49/148 (0.33)	28 (13–50)	66 (57–74)	0.82 (0.42–1.61)	1.09 (0.85–1.41)	14 (6–28)	82 (73–89)	0.47 (0.35–0.59)
Tenderness to palpation								
Lateral	135/150 (0.90)	88 (69–97)	10 (5–17)	0.97 (0.84–1.13)	1.19 (0.35–4.00)	17 (11–25)	80 (51–95)	0.49 (0.37–0.61)
Medial	82/150 (0.55)	38 (21–59)	42 (33–51)	0.66 (0.40–1.10)	1.47 (1.06–2.03)	12 (6–22)	76 (64–86)	0.40 (0.28–0.52)
Anterior	47/150 (0.32)	23 (10–44)	67 (58–75)	0.70 (0.33–1.47)	1.15 (0.92–1.43)	13 (5–26)	81 (71–87)	0.45 (0.33–0.57)
Posterior	19/150 (0.13)	12 (3–31)	87 (80–92)	0.89 (0.28–2.85)	1.02 (0.88–1.17)	16 (4–40)	82 (75–88)	0.49 (0.37–0.61)
Tenderness length [≥ 2.5 cm]	69/139 (0.50)	67 (45–84)	54 (44–63)	1.45 (1.02–2.04)	0.62 (0.35–1.10)	23 (14–35)	89 (78–95)	0.60 (0.48–0.73)
Normal walk	77/150 (0.51)	62 (41–79)	51 (42–60)	1.25 (0.88–1.78)	0.76 (0.46–1.25)	21 (13–32)	86 (76–93)	0.56 (0.44–0.68)
Walk on toes	89/147 (0.61)	69 (48–85)	41 (33–51)	1.18 (0.88–1.59)	0.74 (0.41–1.36)	20 (13–30)	86 (74–93)	0.55 (0.43–0.67)
Walk on heels	79/144 (0.55)	56 (35–75)	45 (36–55)	1.03 (0.70–1.51)	0.97 (0.61–1.54)	18 (10–28)	83 (71–91)	0.51 (0.38–0.63)
Range of motion								
Pain on dorsal flexion	94/150 (0.63)	58 (37–76)	36 (28–45)	0.90 (0.63–1.29)	1.17 (0.72–1.88)	16 (9–25)	80 (67–89)	0.47 (0.35–0.59)
Pain on plantar flexion	83/150 (0.55)	56 (35–75)	43 (35–53)	0.99 (0.68–1.45)	1.01 (0.64–1.61)	17 (10–27)	83 (71–91)	0.50 (0.37–0.62)
Pain on inversion	99/150 (0.66)	69 (48–85)	34 (26–43)	1.05 (0.79–1.40)	0.90 (0.49–1.65)	18 (11–27)	84 (70–92)	0.52 (0.40–0.64)
Pain on eversion	85/150 (0.56)	56 (35–75)	42 (33–50)	0.95 (0.65–1.39)	1.06 (0.67–1.70)	16 (10–26)	82 (70–90)	0.49 (0.36–61)
Presence of swelling								
Lateral	102/131 (0.78)	88 (67–97)	24 (17–34)	1.16 (0.96–1.39)	0.51 (0.17–1.57)	21 (13–30)	90 (72–97)	0.56 (0.44–0.68)
Medial	47/131 (0.36)	38 (20–59)	64 (55–73)	1.06 (0.59–1.88)	0.97 (0.70–1.33)	19 (10–34)	82 (72–89)	0.51 (0.38–0.64)
Anterior	37/131 (0.28)	25 (11–47)	71 (61–79)	0.86 (0.41–1.83)	1.06 (0.83–1.34)	16 (7–33)	81 (71–88)	0.48 (0.35–0.61)
Posterior	12/131 (0.09)	8 (1–28)	91 (83–95)	0.89 (0.21–3.81)	1.01 (0.89–1.14)	17 (3–49)	82 (73–88)	0.50 (0.37–0.62)

The prevalence of acute syndesmosis injuries and the diagnostic values of clinical findings are presented. All values are presented with 95% confidence interval (95% CI); positive likelihood ratio (LR+); negative likelihood ratio (LR-); positive predictive value (PPV); negative predictive value (NPV)

**Table 3:** Diagnostic accuracy of six syndesmosis tests and clinical suspicion for injury of the syndesmosis ligaments in 150 athletes with an acute ankle injury. MR imaging was used as reference standard

	Positive findings	Sensitivity %	Specificity %	LR+	LR−	PPV %	NPV %	AUC
Syndesmosis tests								
Tenderness AITFL	78/150 (0.52)	58 (37–76)	49 (40–58)	1.14 (0.78–1.65)	0.86 (0.54–1.37)	19 (12–30)	85 (74–92)	0.52 (0.43–0.61)
Squeeze test	38/150 (0.25)	46 (27–66)	79 (71–86)	2.20 (1.29–3.77)	0.68 (0.48–0.98)	32 (18–49)	88 (80–93)	0.60 (0.49–0.71)
NWB DF ER test	74/148 (0.50)	58 (37–76)	52 (42–61)	1.19 (0.82–1.74)	0.82 (0.52–1.30)	20 (12–32)	85 (75–92)	0.53 (0.44–0.62)
WB DF ER test	99/149 (0.66)	69 (48–85)	34 (26–43)	1.05 (0.79–1.40)	0.90 (0.49–1.65)	18 (11–27)	84 (70–92)	0.51 (0.42–0.61)
Fibular translation test	26/147 (0.18)	15 (5–36)	82 (74–88)	0.85 (0.32–2.25)	1.03 (0.87–1.22)	15 (5–36)	82 (74–88)	0.49 (0.37–0.61)
Cotton test	21/139 (0.15)	27 (12–48)	88 (80–93)	2.17 (0.98–4.84)	0.83 (0.66–1.06)	33 (15–57)	84 (76–90)	0.59 (0.45–0.73)
All tests negative	35/147 (0.23)	23 (10–44)	76 (67–83)	0.96 (0.45–2.08)	1.01 (0.82–1.26)	17 (7–34)	82 (74–88)	0.50 (0.37–0.62)
Clinical suspicion								
Syndesmosis injury	79/143 (0.55)	73 (52–88)	49 (39–58)	1.43 (1.06–1.91)	0.55 (0.29–1.06)	24 (15–35)	89 (78–95)	0.61 (0.49–0.73)

The prevalence of acute syndesmosis injuries and the diagnostic values of six syndesmosis tests (including the combination of these six tests) and clinical suspicion are presented. All values are presented with 95% confidence interval (95% CI); positive likelihood ratio (LR +); negative likelihood ratio (LR-); positive predictive value (PPV); negative predictive value (NPV)

### Univariate analysis

Various predictors were associated with injury of the syndesmosis ligaments in univariate logistic regression analysis. (Table 4). Eversion mechanism of injury (OR 4.57; CI 95% 1.62–12.89; *p* < 0.00), a positive squeeze test (OR 3.23; CI 95% 1.34–7.82; *p* = 0.01), tenderness length [≥ 2.5 cm] (2.34; CI 95%0.93–5.90; *p* = 0.07) and clinical suspicion (OR 2.58; 1.01–6.60; *p* = 0.05) were associated with higher odds of syndesmosis injury. Inversion mechanism of injury (OR 0.26; 0.10–0.69; *p* = 0.01) was associated with lower odds of syndesmosis injury.

**Table 4:** Univariate logistic regression analysis for the association between injury history, physical examination, syndesmosis tests and clinical suspicion for the presence of syndesmosis injury

	*N*	OR (95%CI)	SE	*P*-value
Clinical history				
Injury [recurrent]	150	0.35 (0.10–1.23)	0.65	**0.10**
Occasion [game]	150	1.32 (0.56–3.10)	0.44	n.s
Contact [contact]	138	0.57 (0.22–1.46)	0.49	n.s
Mechanism of injury				
Inversion	150	0.26 (0.10–0.69)	0.50	**0.01**
Eversion	150	4.57 (1.62–12.89)	0.53	**0.00**
External rotation	150	5.08 (0.68–37.87)	1.03	**0.11**
Internal rotation	150	2.50 (0.43–14.43)	0.89	n.s
Perceived swelling	149	1.01 (0.31–3.25)	0.60	n.s
Pain radiating up	146	1.05 (0.38–2.89)	0.52	n.s
Clinical findings				
Presence of hematoma	148	0.75 (0.29–1.94)	0.48	n.s
Tenderness to palpation				
Lateral	150	0.82 (0.22–3.14)	0.69	n.s
Medial	150	0.45 (0.19–1.07)	0.44	**0.07**
Anterior	150	0.61 (0.23–1.63)	0.50	n.s
Posterior	150	0.85 (0.24–3.27)	0.67	n.s
Tenderness length [≥ 2.5 cm]	139	2.34 (0.93–5.90)	0.47	**0.07**
Normal walk	150	1.65 (0.70–3.93)	0.44	n.s
Walk on toes	147	1.59 (0.64–3.93)	0.46	n.s
Walk on heels	144	1.06 (0.44–2.52)	0.44	n.s
Range of motion				
Pain with passive dorsal flexion	150	0.77 (0.33–1.84)	0.44	n.s
Presence of swelling	142	1.26 (0.34–4.67)	0.67	n.s
Lateral	131	2.25 (0.62–8.15)	0.66	n.s
Medial	131	1.09 (0.44–2.72)	0.47	n.s
Anterior	131	0.82 (0.30–2.25)	0.52	n.s
Posterior	131	0.88 (0.18–4.31)	0.81	n.s
Syndesmosis tests				
Tenderness AITFL	150	1.32 (0.56–3.10)	0.44	n.s
Squeeze test	150	3.23 (1.34–7.82)	0.45	**0.01**
NWB DF ER test	148	1.00 (0.98–1.01)	0.01	n.s
WB DF ER test	149	1.00 (0.97–1.02)	0.01	n.s
Fibular translation test	147	0.82 (0.26–2.61)	0.59	n.s
Cotton test	139	1.00 (0.99–1.00)	0.00	n.s
All tests clinical tests negative	147	0.92 (0.35–2.60)	0.51	n.s
Clinical suspicion				
Syndesmosis injury	143	2.58 (1.01–6.60)	0.48	**0.05**

The odds ratios of the independent variables associated with syndesmosis injury are presented. Values are presented as *β*-coefficients with corresponding 95% confidence interval (95% CI); Standard Error (SE); *P*-value > 0.15 (n.s.)

### Multivariate analysis

In the multivariate logistic regression analysis mechanism of injury [eversion] (OR 4.99; CI 95% 1.56–16.01; *p* = 0.01) and a positive squeeze test (OR 3.25; CI 95% 1.24–8.51); *p* = 0.02) were associated with higher odds of syndesmosis injury (Table 5).

**Table 5:** Multivariate logistic regression analysis for the association between injury history, physical examination and syndesmosis tests for the presence of syndesmosis injury

	***N***	Multivariate		
		OR (95%CI)	SE	*P*-value
Clinical history				
Mechanism of injury [eversion]	150	4.99 (1.56–16.01)	0.60	0.01
Physical examination				
Tenderness to palpation [medial]	150	0.32 (0.12–0.83)	0.49	0.02
Syndesmosis tests				
Squeeze test	150	3.25 (1.24–8.51)	0.49	0.02

The odds ratios of the independent variables associated with syndesmosis injury are presented. Values are presented as *β*-coefficients with corresponding 95% confidence interval (95% CI); Standard Error (SE)

## Discussion

The most important finding in this study is that despite high levels of pain in the acute clinical setting, a negative overall clinical suspicion reduces the probability of syndesmosis injury. An eversion mechanism of injury and a positive squeeze test are associated with higher odds of syndesmosis injury. None of the included variables had sufficient diagnostic value to completely rule out syndesmosis injury.

### Diagnostic value of injury history

Injury history is of major importance for the diagnosis of syndesmosis injury as demonstrated in this study. With an external rotation mechanism of injury the probability of syndesmosis injury increased from a pre-test probability of 17% to a post-test probability of 49%. An eversion mechanism of injury changed the post-test probability to 42%. Despite high specificity, external rotation did not reach significance in the logistic regression analyses as only few patients reported this mechanism of injury. A previous study investigating the diagnostic value of the mechanism of injury reported a positive likelihood ratio of 1.07 (CI 95% 0.87–1.32) with an external rotation and/or dorsiflexion mechanism [22]. As the study by Sman et al. only included patients with at least one positive syndesmosis test or a clinical suspicion of syndesmosis injury selection bias may have occurred. Based on our results we emphasize the importance of history taking and the mechanism of injury in particular [5, 11, 25].

### Diagnostic value of physical examination

The test characteristics of physical examination were insufficient to completely rule out syndesmosis injury in the acute setting. The presence of tenderness over the syndesmosis [≥ 2.5 cm] resulted in a post-test probability of 23%. While peri-ligamentous edema of the syndesmosis was considered disease negative, it is an important source of tenderness and might therefore have had a negative effect on the diagnostic value [6, 14]. Medial tenderness to palpation demonstrated an inverse association in the multivariate logistic regression analysis. Two previous studies reported similar findings [8, 22]. This might be due to the low number of high grade syndesmosis injuries with associated deltoid ligament injury included in these studies [21]. In contrast, compression injury of the deltoid ligaments is commonly observed in inversion injuries [14, 19]. To put our findings into perspective, physical examination of the lateral ankle ligaments within 48 h post-injury has a sensitivity of 71% and a specificity of 33% [6]. Delayed physical examination has a sensitivity of 96% and specificity of 84%. Future studies should therefore aim to investigate the diagnostic value of delayed physical examination for syndesmosis injury.

### Diagnostic value of the individual syndesmosis tests

The squeeze test had the highest discriminative value for the presence of syndesmosis injury in the acute setting. A positive squeeze tests resulted in a post-test probability of 31% and a negative squeeze test resulted in a post-test probability of 12%. None of the six individual syndesmosis tests had sufficient diagnostic value to completely rule out syndesmosis injury. A previous study reported on the diagnostic value of five syndesmosis tests in a cohort of patients presenting to an Emergency department within 24 h post-injury [8]. Of the included tests, the external rotation stress test was found to have the highest sensitvity (56%). It was therefore concluded that clinical tests could not be relied upon. In another study the diagnostic value was investigated in a cohort of athletes examined within 7 days after a suspected syndesmosis injury [22]. The squeeze test was found to have the highest positive likelihood ratio (positive LR 2.15 CI 95% 0.86–5.39). Sensitivity was highest for tenderness over the syndesmosis ligaments (92%) and the non-weight-bearing dorsiflexion external rotation test (71%). Sman et al. therefore advised to combine sensitive tests (tenderness of the syndesmosis ligaments and the dorsiflexion-external rotation stress test) with specific tests (the squeeze tests). As high levels of pain are present in the acute setting, the discrepancy of reported diagnostic values might be attributed to the timing of physical examination. Based on our results, we recommend use of the squeeze test in the acute clinical setting.

### Diagnostic value of clinical suspicion

This is the first study investigating the diagnostic value of clinical suspicion for syndesmosis in the acute clinical setting. Despite high levels of pain, overall clinical suspicion had a sensitivity of 73% and negative predictive value of 89%. When there was no clinical suspicion of syndesmosis injury the post-test probability of syndesmosis injury was reduced to 10%.

The strengths of this study include its prospective design, broad inclusion criteria and acquisition of 3.0-T MR scans within 10 days post-injury [18]. Standardized clinical examination was performed by 19 physicians, trained in 10 different countries. This provides external validity to our findings. To date, this is the largest study evaluating the diagnostic value of injury history, physical examination, syndesmosis tests and overall clinical suspicion for syndesmosis injury. A limitation of this study is that most patients presented to our walk-in clinic within 48 h post-injury. High levels of pain might therefore have affected the reported diagnostic values negatively. Despite our aim to recruit female athletes, only few presented to our walk-in clinic. Although physical examination was standardized and clear test instructions were provided, minor variations in the execution might have influenced the reported diagnostic accuracy. Finally, MRI was used as reference standard despite its inability to diagnose syndesmosis instability [9, 13].

In the acute setting, clinical evaluation may aid in the early recognition of syndesmosis injuries [12]. Clinical evaluation can be used to decide whether to refer for immediate further diagnostic tests or delayed physical examination. Athletes reporting an external rotation mechanism of injury and/or a positive squeeze test might be referred for MRI. Athletes with a negative overall clinical suspicion can be referred for delayed physical examination 5–10 days post-injury. In the acute setting none of the included variables was sufficient to completely rule out the presence of syndesmosis injury. When the aim is to completely rule out syndesmosis injury, diagnostic methods such as (dynamic-) ultrasound might provide a viable alternative [2].

## Conclusion

In an acute clinical setting with patients reporting high levels of ankle pain, a negative overall clinical suspicion reduces the probability of syndesmosis injury. An eversion mechanism of injury and a positive squeeze test are associated with higher odds of syndesmosis injury.

## Supplementary Information

Below is the link to the electronic supplementary material.

Supplementary file1 (PDF 4653 KB)

## Abbreviations

AITFL: Anterior inferior tibiofibular ligament
AUC: Area under the curve
CI: Confidence interval
IOL: Interosseous ligament
IQR: Interquartile range
K: Kappa
LR−: Negative likelihood ratio
LR+: Positive likelihood ratio
MRI: Magnetic resonance imaging
MSK: Musculoskeletal
NPV: Negative predictive value
NWB DF ER: Non-weight-bearing dorsal flexion external rotation
OR: Odd ratio
PD-FS: Proton-density fat-saturated
PITFL: Posterior tibiofibular ligament
PPV: Positive predictive value
T: Tesla
VAS: Visual analog scale
WB DF ER: Weight-bearing dorsal flexion external rotation
