# Supplementary material for: Acute clinical evaluation for syndesmosis injury has high diagnostic value
Source: Knee Surg Sports Traumatol Arthrosc. 2022 May 4;30(11):3871–80. doi: 10.1007/s00167-022-06989-2 (PMC9568458; doi:10.1007/s00167-022-06989-2)
Supplement: Supplementary file 1 — Supplementary file1 (PDF 4653 KB) [file 167_2022_6989_MOESM1_ESM.pdf]

Supplementary appendix description of the six included syndesmosis tests

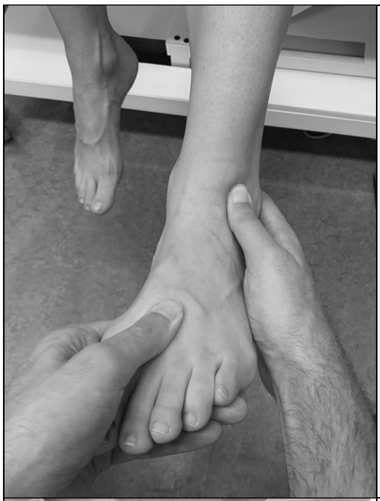	***Palpation of the AITFL*** [4]<br>- *Palpation over the AITFL*<br>- *Positive if pain over the syndesmosis ligaments*
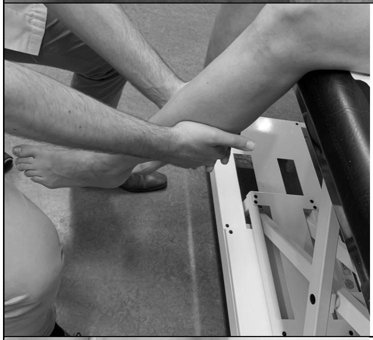	***Squeeze test*** [4,8]<br>- *Patient sitting over the side of the bed. Compression of fibula to the tibia above the midpoint of the calf*<br>- *Positive if pain over the syndesmosis ligaments*
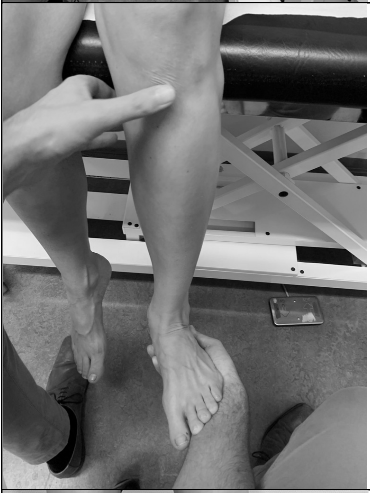	***Non-weight-bearing dorsiflexion external rotation*** [4,9]<br>- *External rotation stress to affected foot and ankle with the knee in 90® and ankle passively in maximal dorsiflexion*<br>- *Positive if pain over the syndesmosis ligaments*
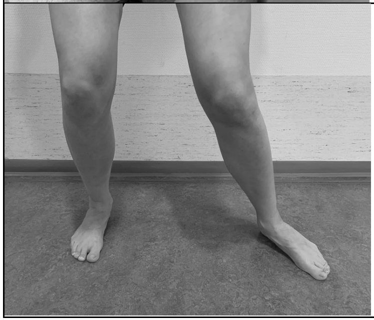	***Weight-bearing dorsiflexion external rotation***<br>- *Active external rotation of the affected foot and ankle in weight-bearing position, with the knee in approximately 30® flexion.*<br>- *Positive if pain over the syndesmosis ligaments*

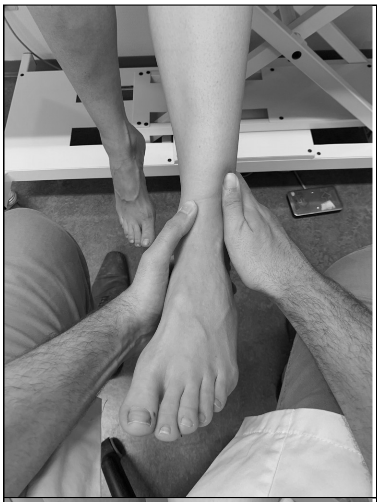	***Fibular translation test*** [10]<br>- *Apply anterior-posterior translation of the fibula*<br>- *Positive if anteroposterior displacement of the fibula is greater than contralateral side, or with pain over the syndesmosis ligaments*
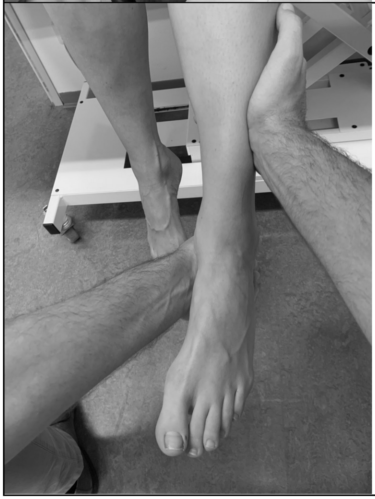	***Cotton test*** [11]<br>- *Distal tibia stabilised and lateral force applied to the foot*<br>- *Positive if increased lateral translation of the talus from medial to lateral compared with contralateral side*
